# TIFA, an inflammatory signaling adaptor, is tumor suppressive for liver cancer

**DOI:** 10.1038/oncsis.2015.30

**Published:** 2015-10-26

**Authors:** W Shen, A Chang, J Wang, W Zhou, R Gao, J Li, Y Xu, X Luo, R Xiang, N Luo, D G Stupack

**Affiliations:** 1 Department of Immunology, School of Medicine, Nankai University, Tianjin, China; 2 Department of Reproductive Medicine, San Diego School of Medicine, University of California, San Diego, San Diego, CA, USA

## Abstract

TIFA (TNF receptor associated factor (TRAF)-interacting protein with a Forkhead-associated (FHA) domain), also called T2BP, was first identified using a yeast two-hybrid screening. TIFA contains a FHA domain, which directly binds phosphothreonine and phosphoserine, and a consensus TRAF6-binding motif. TIFA-mediated oligomerization and poly-ubiquitinylation of TRAF6 mediates signaling downstream of the Tumor necrosis factor alpha receptor 1 (TNFaR-I) and interleukin-1/Toll-like receptor 4 (TLR4) pathways. Examining TIFA expression in hepatocellular carcinoma (HCC) tissues microarrays, we noted marked decreases TIFA reactivity in tumor versus control samples. In agreement, we found that HCC cell lines show reduced TIFA expression levels versus normal liver controls. Reconstituting TIFA expression in HCC cell lines promoted two independent apoptosis signaling pathways: the induction of p53 and cell cycle arrest, and the activation of caspase-8 and caspase-3. In contrast, the expression of a non-oligomerizing mutant of TIFA impacted cells minimally, and suppression of TIFA expression protected cells from apoptosis. Mice bearing TIFA overexpression hepatocellular xenografts develop smaller tumors versus TIFA mutant tumors; terminal deoxynucleotidyl transferase dUTP nick end labeling staining demonstrates increased cell apoptosis, and decreased proliferation, reflecting cell cycle arrest. Interestingly, p53 has a greater role in decreased proliferation than cell death, as it appeared dispensable for TIFA-induced cell killing. The findings demonstrate a novel suppressive role of TIFA in HCC progression via promotion of cell death independent of p53.

## Introduction

Chronic liver inflammation is associated with increased incidence of liver cancer. Hepatocellular carcinoma (HCC), the most common liver cancer, is an end product of chronic liver disease typically requiring decades to evolve. HCC is the fifth most common cancer worldwide, with a doubling incidence in the United States alone during the last two decades.

An increased incidence of inflammatory mediators, such as ATF4,^1, 2^ TLR4,^3, 4^ TREM-1,^5, 6^ have been linked to the development of HCC; other proteins, such as TIFA (TRAF-interacting protein with a Forkhead-associated (FHA) domain), are upregulated in response to hypoxia or other acute stress.^7^ The most frequent cause for chronic hepatic inflammation in humans is infection with hepatitis B virus or hepatitis C virus, which currently persist in approximately 500 million people worldwide, and fosters an increasing HCC patient population. Effective means to eradicate these chronic viral infections have been elusive, although new treatments for hepatitis C virus are poised to make a significant impact if economic barriers can be overcome.

Analysis of fundamental inflammatory signaling pathways may therefore reveal targets or markers to identify and treat patients with chronic liver inflammation, particularly those predictive of HCC. TIFA, also called T2BP, was identified as a TRAF6-interacting protein in a yeast two-hybrid screen,^8^ but may also bind TRAF2.^9^ In addition to the FHA domain, a phosphothreonine and phosphoserine-binding motif,^10^ TIFA contains a consensus TRAF6-binding motif.^11, 12^ Elevated TIFA expression activates nuclear factor (NF)-κB and c-JUN N-terminal kinase in a manner dependent on TRAF6,^13^ and links TRAF6 to NF-κB in the interleukin-1/TLR4 pathway.^8^ TIFA is increased after cell stress such as hypoxia; the increase in TIFA level appears to feed forward into TLR4/MyD88-dependent signaling, leading to NF-κB activation and HMGB1 release.^7^ The data are consistent with a pro-tumorigenic role for TIFA.

Here, we studied the role of TIFA in normal liver and HCC. Unexpectedly, we find that TIFA expression is suppressed during tumor progression, in contrast to other inflammatory mediators.^2, 4, 5^ In agreement, TIFA reconstitution induced the expression of p53, promoting apoptosis while suppressing proliferation among surviving cells. The studies implicate TIFA as a previously unappreciated suppressor of liver carcinogenesis via p53-dependent and -independent mechanisms, and provide insight into a vulnerability of HCC.

## Results

### TIFA is decreased in HCC

**Figure 1:**
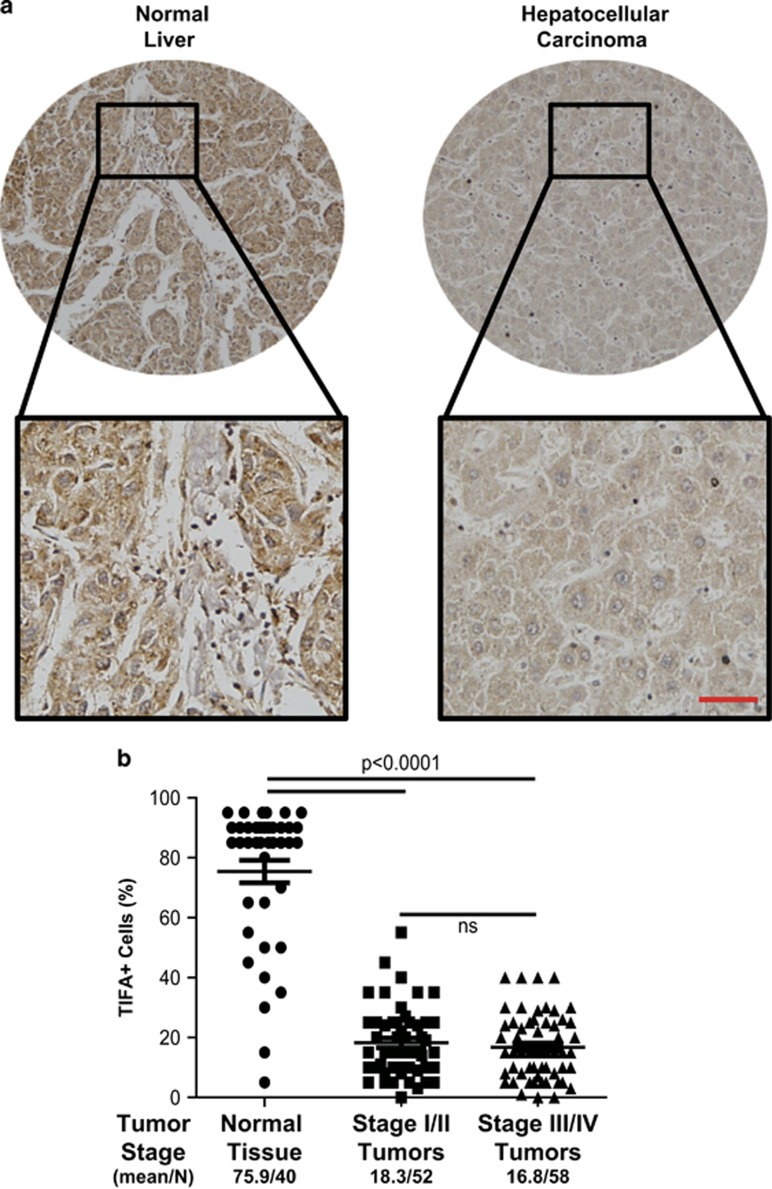
TIFA protein abundance is decreased in HCC tissue. (**a**) The representative image of TIFA staining in human tissue microarrays (including 110 HCC samples and 50 normal samples). Scar bar, 100 μm. (**b**) The relationship of TIFA-positive cells with tissue status or HCC clinical stage. Each point represents one sample and the middle line represents the mean value. Data are shown as the mean±s.e.m. The number of samples and the mean values of each group are listed below the x axis. (*P*-value was determined by Student's *t*-test).

The expression of TIFA was examined in microarrays containing liver biopsies from 150 patients (110 HCC samples and 40 normal samples). As shown (Figure 1a), a relatively robust expression of TIFA was detectable in normal liver tissues, whereas in contrast, TIFA expression was weakly detected in frank carcinoma. TIFA loss was notable even in early-stage disease (Figure 1b).

As TIFA has a role in promoting inflammation, which fosters HCC, this result was somewhat unexpected. Therefore, we also examined TIFA protein data presented in the Human Protein Atlas (HPA) (www.proteinatlas.org). Despite using a different antibody to detect TIFA, HPA data were in agreement with our observations; the detection of TIFA was decreased in liver cancer relative to controls (Supplementary Table 1).

### TIFA expression is suppressed in HCC tumor cell lines

**Figure 2:**
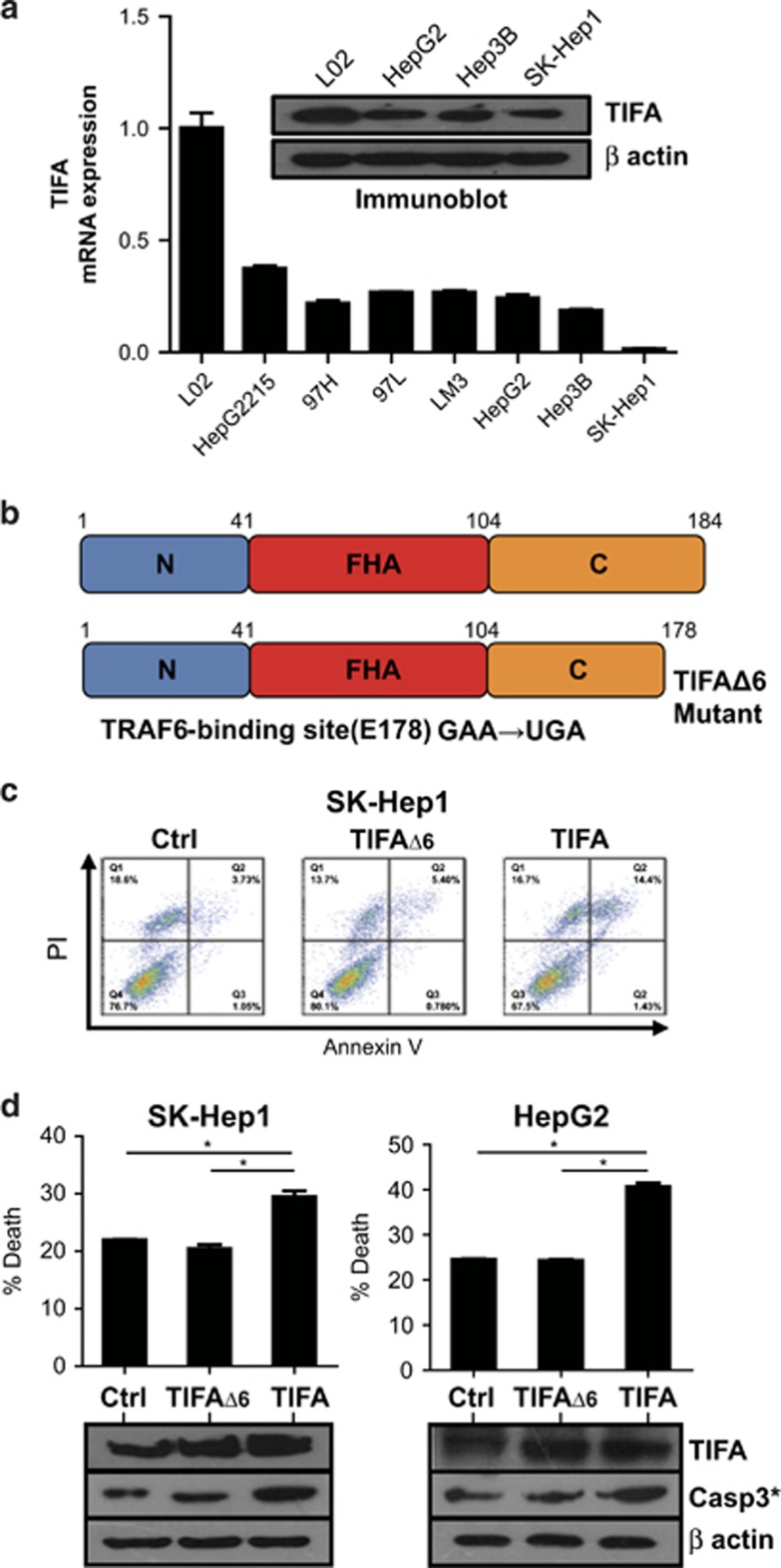
Ectopic expression of TIFA promotes apoptosis in hepatoma cells. (**a**) Real-time PCR and western blot were performed to detect the expression of TIFA in normal liver cell and HCC cell lines. (**b**) Overview of TIFA structure. TIFA contains three domains (N, FHA, C). The E178* mutant (GAA/TGA) lacks the last six amino acids that interact with TRAF6 (TIFAΔ6). (**c**) Flow cytometry showing the percentage of dead cells detected by PI-Annexin V double staining in TIFA or TIFAΔ6 cells. A representative experiment of three is shown. (**d**) Statistical results of the percentage of apoptotic cells are shown. The immunoblotting shows relative TIFA expression as well as the expression of cleaved caspase-3. β-Actin is included as a loading control.

We investigated whether the observations in clinical samples were represented in tumor cell lines. The mRNA level of TIFA was found to be decreased in HCC cell lines relative to normal liver cell line (Figure 2a, inset). Immunoblot analysis of TIFA protein levels supported the quantitative PCR results, suggesting that TIFA was in fact decreased in the cell lines (Figure 2a). Therefore, the cell lines appeared suitable to use as a model to determine the impact of TIFA on HCC biology.

To investigate the functional role of TIFA in HCC, we reconstituted either wild-type (WT) TIFA, or an oligomerization defective (TRAF6-binding mutant, TIFAΔ6) (Figure 2b) into HCC tumor lines. We noted some loss of cells in the TIFA expression groups. Checking more carefully, we noted cell death within the HCC tumor lines SK-Hep-1 (Figures 2c and d) following expression of TIFA. However, expression of TIFAΔ6, which does not bind TRAF6, did not induce cell death. As reported by others, coprecipitation studies supported a selective and constitutive interaction of TIFA with TRAF6^7^ (unpublished data), but not TRAF2, a component of (apoptotic) complex II. Nonetheless, apoptosis was suggested by Annexin-V staining, and was further supported by detection of increased levels of the cleaved form of caspase-3. Similar results were observed with the HepG2 cell line (Figure 2d), supporting the notion that TIFA expression could promote apoptosis among HCC.

### TIFA reconstitution fosters apoptosis *in vivo*

**Figure 3:**
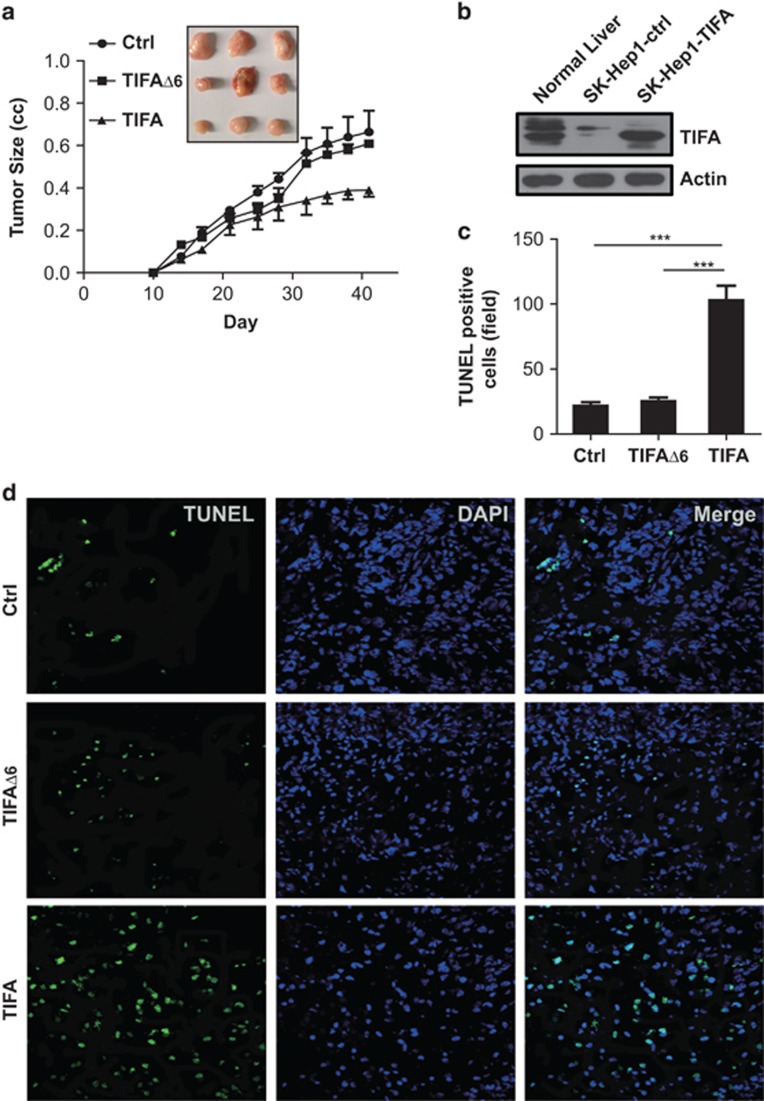
Ectopic expression of TIFA promotes cell apoptosis *in vivo*. (**a**) Tumor growth curve of control, TIFA, and TIFAΔ6 tumors harvested from the flank of NOD/SCID mice. Several tumors separated from each group are shown (inset). (**b**) Immunoblot analysis of the expression of TIFA in reconstituted and control SK-Hep1 cells relative to normal liver. (**c**) Bar graph shows the statistical results of TUNEL staining illustrated in panel **c**. Scar bar, 100 μm. *P<0.05, **P<0.01, ***P<0.001 compared with control by Student's *t*-test. (**d**) Representative images showing TUNEL staining of xenografted tumor tissue from each group (control, TIFA, TIFAΔ6).

To determine if the pro-apoptotic effects of TIFA were maintained *in vivo*, we next performed tumor xenograft studies in nude mice. Consistent with the results obtained using *in vitro* assays, a significantly diminished tumor size was observed selectively among tumors from SK-Hep1 TIFA-expressing cells, relative to controls or SK-Hep1 TIFAΔ6-expressing cells (Figure 3a). The harvested TIFA-expressing tumors exhibited TIFA protein levels similar to that seen in normal liver (Figure 3b). Concordant with the decreased tumor size, we observed increased terminal deoxynucleotidyl transferase dUTP nick end labeling (TUNEL) staining within the tumor proper (Figure 3c). By contrast, the control and TIFAΔ6-expressing tumors grew similarly, and displayed similar levels of TUNEL staining; roughly one-fifth of that seen in TIFA-positive tumors. Thus, TIFA expression was deleterious to HCC *in vivo* as well as *in vitro*. Mechanistically, this was at least partly due to apoptosis.

### TIFA promotes cell cycle arrest

**Figure 4:**
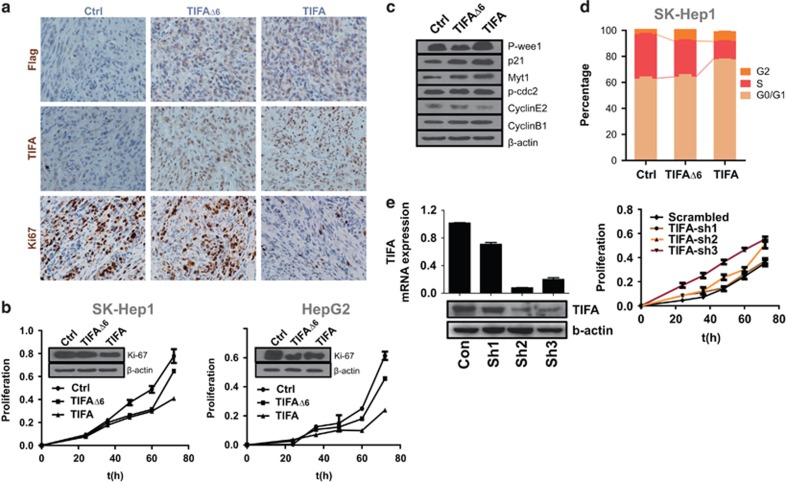
Ectopic expression of TIFA inhibits cell proliferation *in vitro* and *in vivo*. (**a**) The images show the immunohistochemistry staining of FLAG, TIFA and Ki-67 in tumor tissues separated from xenografted NOD/SCID mice subcutaneously injected with control, TIFA or TIFAΔ6 SK-Hep1 cells. Scar bar, 100 μm. (**b**) The cell growth of two cell lines (SK-Hep1 cell and HepG2 cell) were analyzed at 0, 24, 48, 60 and 72 h after cell seeding. Cell lysates were immunoblotted with antibodies detecting the ki-67 using specific anti-Ki-67 antibody are shown in the inset. β-Actin was used as a loading control. (**c**) Immunoblot showing cell cycle-related protein expression in TIFA or TIFAΔ6 cells. β-Actin is included as a loading control. (**d)** Stacked bar graph showing the result of three separated experiments of cell cycle analysis via 7-aminoactinomycin D (7-AAD)/5-bromo-2-deoxyuridine (BrdU)-FITC double staining analyzed by flow cytometry. (**e**) Lentiviral expression of shRNA to TIFA was evaluated by immunoblotting for effect in SK-HEP1 cells (right panel), and for effect on cell proliferation (left panel).

We also evaluated proliferation via an assessment of Ki-67 staining to control for cell proliferation as an alternative explanation for the disparity in tumor size. Unexpectedly, TIFA expression was associated with significantly decreased Ki-67 reactivity in tumors, indicating that apoptosis may not act alone to limit tumor size (Figure 4a). Evaluating growth *in vitro*, we confirmed that TIFA expression led to differences of both overall proliferation in SK-Hep1 and HepG2 cells (Figure 4b), most particularly with long-term culture proliferation. The difference in net proliferation did not appear to be explainable by cell loss because of apoptosis, and indeed there was a concomitant increase in Myt1/CDC2 as well as p21, with a decrease in cyclin E2 (Figure 4c). Moreover, it was accompanied by an increase in the G0/G1 population of cells (Figure 4d). Conversely, suppression of TIFA via short hairpin RNA (shRNA)-mediated silencing promoted proliferation (Figure 4e). The collective evidence suggested that TIFA expression acted to suppress tumor progression via more than one mechanism.

**Figure 5:**
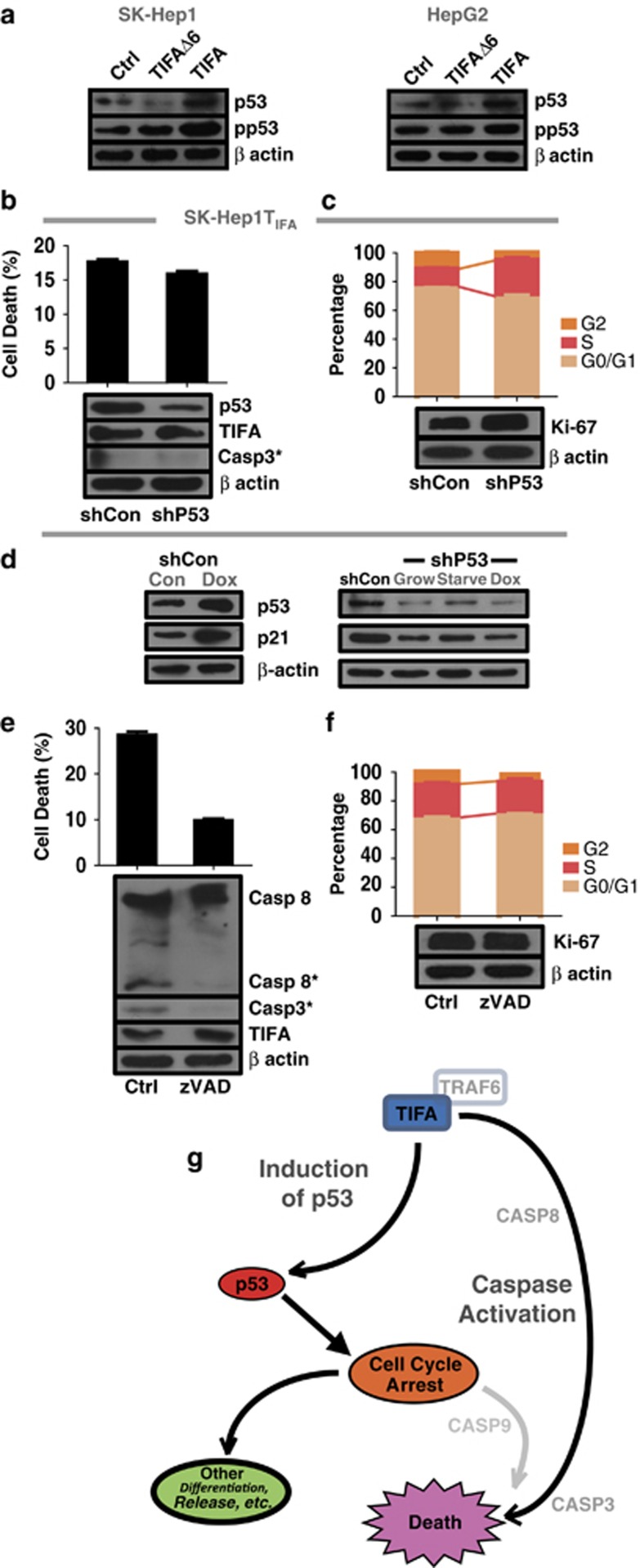
TIFA-mediated apoptosis depends on caspase activation, but not p53 accumulation. (**a**) Western blot was performed to analysis p53 activation in TIFA or TIFAΔ6 cells using specific anti-p53 and anti-phospho-p53 antibody. β-Actin was used as a loading control. (**b**) The bar graph shows the result of three separate experiments examining percentage of cell death after p53 suppression. Immunoblot detects cleaved caspase-3 expression in p53 suppressed cells using specific active/mature anti-caspase-3. β-Actin was included as a loading control. (**c**) 5-Bromo-2-deoxyuridine (BrdU)-FITC and 7-aminoactinomycin D (7-AAD) double staining was used to analysis cell cycle with p53 silence down cells. Representative statistical result was shown. The corresponding result of ki-67 expression was measured by western blot. (**d**) Immunoblotting of shCon cells (left panel) or shP53 cells (right panel) showing the expression of p53 and p21 in cell culture (Grow), upon the addition of Doxarubicin in plain media (1 ug) for 24 h (Dox), or control plain media (Starve). (**e**) The bar graph shows the statistics result of three separate experiments regarding percentage of cell death of SK-Hep1 TIFA cells treated with Z-VAD. Immunoblotting shows mature and cleaved forms of caspase-8 as well as cleaved caspase-3. β-Actin was included as a loading control. (**f**) BrdU-FITC and 7-AAD double staining was used to detect cell cycle after cell treated with Z-VAD. Representative statistics result was shown. The corresponding result of ki-67 expression was measured by western blot. (**g**) Possible model of TIFA in HCC. Reconstituting TIFA expression in HCC cell lines promoted two independent effects; the activation of caspases (3 and 8) possibly analogous to activation via classic complex 2 (TRADD/TRAF2) by TNFa or TLRs, as well as the induction of p53 and cell cycle arrest.

In agreement with this, a parallel Pathscan Array study showed that, relative to controls, an enhanced level of c-JUN N-terminal kinase and phospho-p53 was noted in TIFA-expressing cells (unpublished observations). As p53 activation fosters cell cycle arrest and apoptosis,^14^ and microRNA that targets TIFA can downregulate p53 and Bak, we next evaluated a role for p53 downstream of TIFA in the HCC cells. Immunoblot analysis of p53 protein expression revealed that TIFA expression in either Sk-Hep1 or HepG2 lead to an enhancement of both overall p53 levels as well as levels of phosphorylated p53 (Ser 15; Figure 5a). The observation was consistent with both increased apoptosis observed and cell cycle arrest.

### TIFA acts via p53-dependent and -independent mechanisms

To further test whether p53 may be a key element downstream of TIFA, we next used an shRNA approach to stably suppress p53 expression among cells reconstituted for TIFA expression. Perhaps surprisingly, the loss of p53 did not rescue apoptosis induced by TIFA expression (Figure 5b). However, amelioration of the cell cycle block and proliferation was observed (Figure 5c), suggesting that p53, while required for cell cycle arrest, was not essential for apoptosis in this system. Concordant with this, low levels of p21 induction (relative to controls) were detected in the p53 suppressed cells (Figure 5d).

We next examined the mechanism by which these cells undergo apoptosis via an examination of the initiator and downstream caspases. In addition to the executioner caspase-3, we detected cleavage of procaspase-8 (but not 9) associated with its maturation and activation. The addition of caspase inhibitors blocked caspase-8 cleavage and rescued HCC cell apoptosis (Figure 5e), but did not significantly impact the cell cycle (Figure 5f). The results implicate a p53-independent mechanism by which TIFA induces cell death, and a p53-dependent mechanism of cell cycle arrest (Figure 5g). As >30% of HCC may lose p53 function, the direct activation of caspase activity may represent a key mechanism to limit tumorigenesis.

## Discussion

HCC is the third most common cause for cancer-related death worldwide.^15^ In some African and Asian countries, HCC has become the most prevalent cause for cancer-related death.^16^ Considering the effort invested, the clinical success to pharmacologically treating HCC patients has been limited, although the development of antivirals, such as Sofosbuvir, is promising. A better understanding of tumorigenesis, and the subsequent analysis and identification of new targets or predictive markers for HCC therapy is timely and, for many, urgent.

The roles shown for here for TIFA are unexpected. Most inflammatory mediators tend to promote HCC development. TIFA, by contrast, is not observed to be upregulated in HCC. In fact, this is independent of tumor stage, supporting the notion that loss or suppression of TIFA may be an early step toward tumor development. Indeed, TIFA can foster HCC apoptosis, and loss of apoptotic sensitivity is a hallmark of cancer.^17^

Caspase-1 and 2, in particular, are implicated in inflammation,^18, 19, 20^ and caspase inhibitors targeting these are in clinical development.^21, 22^ Suppression of inflammation may, however, carry an oncogenic risk, if early events fostering tumor progression are already in process, and the caspase-dependent killing we observe is prevented. This may be particularly important in the generation of the p53-compromised subgroup of HCC (30–40%) where caspase activity may be a final barrier to malignancy.

TIFA has been associated with other signaling pathways, most notably, the NF-κB pathway,^9, 11, 13^ which promotes oncogenesis and tumor progression via nuclear translocation and activation of target genes. TIFA expression can also promote activation of the mitogen-activated protein kinase, extracellular signal-regulated kinase, c-JUN N-terminal kinase and p38. Activation of c-JUN N-terminal kinase and p38 in particular can trigger downstream cascades that lead to inflammation, differentiation or cell death.^23, 24, 25, 26, 27, 28^ Although we observed some activation of these pathways in both the SK-Hep1 and the HepG2 cell lines following TIFA expression (unpublished observations), the patterns and role of these activations were not simple or clear, and further manipulation of these pathways is ongoing to determine if they are required or even relevant to the processes described here.

The studies here present two unexpected findings. The first is that the protein TIFA, which fosters NF-κB signaling,^13^ can induce apoptosis and thus may have a novel role as a regulator of HCC development by fostering removal of transformed cells. This function of TIFA requires the six C-terminal amino-acid residues that bind TRAF6. The second is that this activity proceeds concordant with p53-mediated induction of cell cycle arrest, but occurs even when p53 expression is suppressed. We propose that the oligomerization-competent form of TIFA is poised to promote caspase-dependent cell death as a counterpoint to ongoing inflammation. Caution may be warranted in the development of caspase inhibitors as a mechanism of combating chronic liver inflammation.

## Materials and methods

### Vector construction

The complementary DNA of human TIFA was cloned by reverse transcription PCR using total RNA from L02 cells, and using the specific primer pairs for TIFA (forward: 5′-CGGGATCCATGACCAGTTTTGAAGATGCTG-3′, reverse: 5′-CGGAATTCTCATGACTCATTTTCATCCATTTC-3′). The amplified fragments were first ligated into pCMV-Tag 2B labeled with FLAG tag and then cloned using the primer pairs for Flag-TIFA (forward: 5′-GCTCTAGAGCCACCATGGATTACAAGGAT-3′, reverse: 5′-CGACGCGTTCATGTCGGAGAACTGCTTTG-3′). The amplified fragments were finally ligated into pLV-EF1α-MCS-IRES-Bsd (cat. #cDNA- pLV 03, Biosettia Inc., San Diego, CA, USA) expression vector. The mutant TIFA fragments were cloned from the overexpression vector with using the primer pairs for TIFAΔ6. The amplified fragments were also finally ligated into pLV-EF1α-MCS-IRES-Bsd (cat. #cDNA- pLV 03, Biosettia Inc.) expression vector. The shRNA sequence for silencing mice *TIFA* gene was searched and blasted using RNAi designer from the Invitrogen website (https://rnaidesigner.invitrogen.com/rnaiexpress/index.jsp). ShRNA targeting human *TIFA* and scrambled control sequence were designed and chemically synthesized as TIFA–shRNA1 (5′-AAAAGGTCAGATTCGGAGAGTATCATTGGATCCAATGATACTCTCCGAATCTGACC-3′) TIFA–shRNA 2 (5′-AAAAGGTGAAATTTGGCCGAAATTCTTGGATCCAAGAATTTCGGCCAAATTTCACC-3′) and TIFA-shRNA3 (5′-AAAACCAAGATCACTCTTGCAAGAATTGGATCCAATTCTTGCAAGAGTGATCTTGG-3′). The control, TIFA-sc, was 5′-AAAAGCTACACTATCGAGCAATTTTGGATCCAAAATTGCTCGATAGTGTAGC-3′. The palindromic DNA oligos were annealed to each other to form a double-strand oligo and ligated to the linearized pLV-H1-EF1α-puro (cat. #SORT-B19, Biosettia Inc.) vector to generate circled pLV-EF1α-shRNA-TIFA-Puro.

### Cell culture

Human normal liver cell line, L02, was obtained from the Chinese Academy of Sciences (Beijing, China). L02 cell line was maintained in RPMI-1640 media supplemented with 10% fetal bovine serum, 100 U/ml penicillin/streptomycin. Human HCC cell line, wt of SK-Hep1 cell line was kindly provided by Dr Ralph A Reisfeld (The Scripps Research Institute, La Jolla, CA, USA). WT of HepG2 was obtained from the Chinese Academy of Sciences. SK-Hep1-Wt and HepG2-Wt cells were infected with lentivirus carrying pLV-EF1α-Flag-TIFA-IRES-Bsd and pLV-EF1α-Flag-TIFAΔ6-IRES-Bsd plasmids, followed by clonal selection using Blasticidin (5 μg/ml for SK-Hep1 and HepG2) to generate polyclonal cell populations with stable overexpression of Flag-TIFA and TIFAΔ6 (Sk-Hep1-Flag-TIFA, Sk-Hep1-Flag-TIFAΔ6 and HepG2-Flag-TIFA, HepG2-Flag-TIFAΔ6). Alternatively, SK-Hep1-Wt cells were infected with lentivirus carrying pLV-H1-shp53-puro or pLV-H1-shTIFA-puro plasmid, followed by clonal selection using 1 μg/ml puromycin to generate polyclonal cell populations with stable expression of shp53 or shTIFA. For control purposes, SK-Hep1-Wt and HepG2-Wt were infected with lentivirus carrying the empty vector plasmid, SK-Hep1-Wt was infected with lentivirus carrying scrambled shRNA plasmid (see below for sequence) and subjected to identical clone selection procedures to generate the stable control cell lines SK-Hep1-Ctrl, HepG2-Ctrl and SK-Hep1-SC. SK-Hep1 and HepG2 cells were maintained in Dulbecco's modified Eagle's media supplemented with 10% fetal bovine serum, 100 U/ml penicillin/streptomycin and 1% nonessential amino acids.

### Real-time reverse transcriptase–PCR

Total mRNAs from different cell lines were isolated by TRIzol reagent (cat. #15596-018, Invitrogen Inc., Carlsbad, CA, USA) and reverse transcribed into complementary DNAs with MMLV reverse transcriptase (Promega, Madison, MI, USA). Real-time PCR was performed on Opticon (Bio-Rad, Hercules, CA, USA) in 20 μl reaction volumes by using TransStart Green qPCR SuperMix Kit (TransGen Biotech, Beijing, China). The 2^−ΔΔCt^ method was used to determine the relative mRNA folding changes. Statistical results were averaged from three independent experiments performed in triplicate.

### Immunoblotting

Cell lysates from different cell lines were prepared with RIPA buffer in the presence of protease inhibitor cocktails and Phosphatase Inhibitor Cocktail 2 and 3 (P8340, P5726 and P0044, Sigma-Aldrich, St Louis, MO, USA). Protein (30 μg) was loaded onto 5–12% Tris-acrylamide gels and blotted with antibodies that included: anti-TIFA, (cat. #ab103011 Abcam Biotechnology, Inc., Abcam, Hong Kong), β-actin (cat. #sc-47778, Santa Cruz Biotechnology, Inc., Santa Cruz, CA, USA), caspase-3 (cat. #9665, Cell Signal Technology Inc., Danvers, MA, USA), p53 (cat. #sc-126, Santa Cruz Biotechnology, Inc.), p-p53 (cat. #9284p, Cell Signal Technology Inc.), caspase-8 (cat. #9496s, Cell Signal Technology Inc.) and horseradish peroxidase-conjugated secondary antibodies. Blotting results were detected by an ECL chemiluminescence kit (cat. #17153, Millipore, Billerica, MA, USA).

### Flow cytometry analysis of cell cycle and apoptosis

Following culture of cells in the absence of fetal bovine serum for 12 h, cells were ‘pulsed' with 10 mM 5-bromo-2-deoxyuridine for 24 h, and cell cycle assay was performed by using the Cytofix/cytoperm kit (BD Biosciences, San Jose, CA, USA) following the manufacturer's instructions. For apoptosis assay, apoptotic cells were stained with propidium iodide and Annexin-V-FITC (BD Biosciences). Flow cytometry analysis was performed by FACS Calibur cytometer (BD Biosciences), in which a minimum of 10 000 events were recorded. Three independent assays were conducted in such experiments and the mean values were expressed as mean±s.d.

### Tumor xenografts

Male NOD/SCID mice at 6–8 weeks of age were separated randomly into three groups (*n*=5 for each group based on minimal 30% decrease from 1 g tumors with 250 μg s.d. (standard deviation), α error of 0.05 and a β error of 0.8). In all, 3 × 10^6^ SK-Hep1 cells (Sk-Hep1-Flag-TIFA, Sk-Hep1-Flag-TIFAΔ6 and SK-Hep1-Ctrl) were inoculated subcutaneously into each mouse at right axilla. Tumor volume (mm^3^) was measured with calipers two times per week and calculated by using the standard formula: length × width^2^/2. The individual measuring the mice was unaware of the identity of the group measured. Animal use complied with Nankai University Animal Welfare Guidelines.

### Immunohistochemistry

Immunostaining was performed on paraffin human HCC tissue arrays (120 samples from cat. # BC03119, 30 samples from cat. # LV805, Alenabio Company, Shanxi, China). Expression levels of TIFA in the tissue microarray were scored according to the percentage of TIFA-positive cells in each liver tissues. The images were recorded by Olympus BX51 Epi-fluorescent microscopy under a 10 × or 40 × objective (Olympus Co., Tokyo, Japan).

### TUNEL staining

Paraffin-embedded tissue slides were prepared from the tumor xenografts DeadEndTM Fluorometric TUNEL System kit (Promega) was applied for TUNEL staining. Experiment procedure was performed according to the manuscript instruction. 4,6-Diamidino-2-phenylindole was used to stain the nuclei, and the tissue slides were subjected to Olympus BX51 Epi-fluorescent microscopy under a 40 × objective (FV1000-IX81, Olympus Microsystems, Shanghai, China).

### Statistical analysis

Values were expressed as means+s.e.m. Significance was determined by χ^2^ test in Supplementary Table 1, others were determined by Student's *t*-test. A value of *P*<0.05 was used as the criterion for statistical significance. *Indicates significant difference with *P*<0.05, **indicates significant difference with *P*<0.01.
