# Supplementary material for: TIFA, an inflammatory signaling adaptor, is tumor suppressive for liver cancer
Source: Oncogenesis. 2015 Oct 26;4(10):e173–. doi: 10.1038/oncsis.2015.30 (PMC4632091; doi:10.1038/oncsis.2015.30)
Supplement: Supplementary Table 1 [file oncsis201530x1.pdf]

Supplementary Table 1: TIFA staining of HPA-curated liver cancer.

	Patient	Staining Intensity	Subcellullar Localization (If Present)
Tumor	3477	Mosaic (Moderate >> Absent)	N+C
	3841	Mosaic	N+C
	2556	Weak to Absent	N+C
	3324	Absent	
	2339	Diminished	N (*punctate*)
	936	Weak	N
	983	Mosaic (Enhanced/Absent)	N+C
	2177	Mosaic (Moderate/Absent)	N+C
	2766	Diminished	N
	3625	Absent	
	3334	Weak	N
	2279	Weak	N
Control	3222	Moderate	N+C
	3402	Moderate	N+C
	2429	Moderate	N+C

Legend: *Tumor and Control tissues stained for TIFA, curated in the Human Protein Atlas, were examined for the quantity and intensity of staining. Staining pattern was ranked on intensity (Enhanced>Moderate>Diminished>Weak>Absent) and pattern (Uniform is assumed, unless Mosaic is specified). The subcellular localization (nuclear, N, vs cytosolic, C, is specified when the protein was determined to be present). (**www.proteinatlas.org**)*
